# Corrosion Protection of Al/Au/ZnO Anode for Hybrid Cell Application

**DOI:** 10.3390/membranes5040739

**Published:** 2015-11-16

**Authors:** Gymama Slaughter, Brian Stevens

**Affiliations:** Bioelectronics Laboratory, Department of Computer Science and Electrical Engineering, University of Maryland Baltimore County, Baltimore, MD 21250, USA; E-Mail: bstevens1@umbc.edu

**Keywords:** corrosion protection, aluminum activation, oxides, nanostructures, sol–gel growth

## Abstract

Effective protection of power sources from corrosion is critical in the development of abiotic fuel cells, biofuel cells, hybrid cells and biobateries for implantable bioelectronics. Corrosion of these bioelectronic devices result in device inability to generate bioelectricity. In this paper Al/Au/ZnO was considered as a possible anodic substrate for the development of a hybrid cell. The protective abilities of corrosive resistant aluminum hydroxide and zinc phosphite composite films formed on the surface of Al/Au/ZnO anode in various electrolyte environments were examined by electrochemical methods. The presence of phosphate buffer and physiological saline (NaCl) buffer allows for the formation of aluminum hyrdroxide and zinc phosphite composite films on the surface of the Al/Au/ZnO anode that prevent further corrosion of the anode. The highly protective films formed on the Al/Au/ZnO anode during energy harvesting in a physiological saline environment resulted in 98.5% corrosion protective efficiency, thereby demonstrating that the formation of aluminum hydroxide and zinc phosphite composite films are effective in the prevention of anode corrosion during energy harvesting. A cell assembly consisting of the Al/Au/ZnO anode and platinum cathode resulted in an open circuit voltage of 1.03 V. A maximum power density of 955.3 μW/ cm^2^ in physiological saline buffer at a cell voltage and current density of 345 mV and 2.89 mA/ cm^2^, respectively.

## 1. Introductions

Approximately 8% to 10% of Americans and 5% to 6% of the industrialized world’s population have implantable bioelectronic devices [1]. The bioelectronic device implant and biocompatible material market in the United States are 12 and 11 billion dollar industries, respectively. These markets are increasing about 9% every year [2]. Implantable bioelectronics are used for treating diseases, birth defects and the aging population for the overall improvement in the quality of life of the inflicted individuals. This further leads to the reduction of health care costs of the overall population. These implantable bioelectronics range from organ and neuro-stimulators [3] and regeneration devices [4], pacemakers [5,6,7], drug delivery devices [8,9], sensors [10,11,12], and activation circuits that monitor orthopedic implants [13]. These implantable devices could someday be powered by implantable power sources. Most implantable bioelectronics require an internal source of power, which can be accomplished with the help of implantable power sources such as, biobatteries, hybrid cells, biofuel cells and abiotic fuel cells that use abiotic anode or enzymatic anode to catalyze natural occurring fuel inside the body. The Al/Au/ZnO nanostructured anodes have been employed to selectively catalyze glucose in the presence of oxygen in a glucose/ O_2_ fuel cell [14], as well as to generate bioelectricity by activating the Al underneath the ZnO as anodic material in a hybrid cell supplied with phosphate rich electrolyte [15] in the realization of implantable power sources.

In addition, implantable bioelectronic power sources use biomaterials to enhance the biocompatibility, longevity and corrosion protection of the implantable devices from the harsh environments of the body [16,17,18] without adverse side effects. The body is an extremely corrosive environment because of cations such as Na^+^, K^+^, Ca^+^, Mg^+^, anions such as chloride, bicarbonate, phosphate, and large amounts of dissolved oxygen found throughout the body [19,20,21]. These molecules upset the chemical balance on the surface of implanted materials that lead to the consumption of these materials via anodic or cathodic reactions through galvanic corrosion. This corrosion occurs mostly with metallic substrates used to provide structural rigidity and electrical conduction path in bioelectronic devices. The main cause of corrosion comes from oxidation and reduction reactions between metallic materials and biological fluids. In particular, Cl^−^ ions and dissolved oxygen are the major factors in corrosion within the body because of their ability to deteriorate metal substrates. Moreover, implant corrosion can cause serious side effects because of the release of metal anions into the surrounding environments and blood stream. These ions harm the body by either direct toxic effects or local hypersensitivity reactions. Elements such as nickel, cobalt, and chromium as well as their compounds are known allergens [22,23]. Aluminum [24], copper and zinc [25] ions have been associated with Alzheimer’s disease as well as other neurological disorders such as epilepsy. These effects of corrosion from implantable devices has heightened the need for corrosion prevention, especially when harvesting energy from within the body.

Traditionally, corrosion protection is achieved through phosphating using acidic phosphate baths that use nitrates and nitrites. This process has been found to be harmful to the environment [26], whereas anodic acceleration has been employed to produce biocompatible zinc phosphate and zinc phosphate’s hydrated tertiary form, hopeite [27], in physiological conditions using basic neutral pH and buffer solutions. This allows phosphating to occur utilizing biocompatible materials [28,29,30,31,32,33] which not only prevent corrosion but could also generate electricity for biomedical applications. This process can be defined as a biomimetic coating treatment because the process mimics an *in vivo* process for the application of phosphate coatings on an anodic substrate. These biomimetic coatings have also been demonstrated for magnesium substrates by several research groups [34,35], but biomimetic zinc phosphite research has yet to be performed. Here, we explore the application of corrosion prevention for the protection of bioelectronic power sources. Without corrosion protection, bioelectronics devices cease to produce reliable power and can leach harmful metal ions into the body.

In this paper, we take advantage of an economical biomimetic phosphating approach with the ability to form zinc phosphite film in physiological conditions, while generating bioelectricity. The Al/Au/ZnO anode is used for anodic electrochemical treatment in a phosphate rich bath to create corrosion resistive films as well as to produce power for bioelectronic applications. Our approach utilizes a low-cost and “green” alternative method that demonstrates effective corrosion resistance of the anodic substrate. We demonstrate that Al/Au/ZnO anode can be protected in a saline rich solution by the formation of zinc phosphites under physiological conditions rather than using other acidic, high energy and expensive techniques. This technique could be implemented in the body as described by Heller *et al.* [30] as corrosion protection for a bioelectronics power supply for implanted electrical devices.

## 2. Material and Methods

Aluminum foil (99.9999%, 250 mm thick) substrates were cleaned with acetone, isopropanol and deionized water in preparation and fabrication of the Al/Au/ZnO anode using a sol–gel processes [34,35,36]. Magnetron sputtering was used to sputter 40 nm of gold to coat the surface of aluminum substrates. The aluminum (Al) surface activation was achieve via ZnO nanocrystal before zinc phosphating in an Al/phosphate hybrid cell. ZnO precursors were prepared by using 0.4 M zinc chloride (99.99%) and isopropanol. The solution was mixed at 75 °C and equimolar triethenamine was added to stabilize the precursor solution to yield a 0.1 M homogenous ZnO nanosol, which was aged at 85 °C. The ZnO seed layers were deposited on the Al/Au substrate using a dip coating method upon aging of the solution. The solvent was allowed to naturally evaporate followed by annealing at 150 °C for 1 hour. The dip coat method was repeated multiple times to create a uniform seed layer and finally dried at 30 °C in a convection oven for 12 hours [14,15,36].

All current-voltage and power curves were obtained by acquiring the current and voltage through and across a variable load. Device durability tests of the assembled cell were performed with the Al/Au/ZnO anode and a Pt cathode at a load of 3 kΩ, since the maximum power for the hybrid cells were obtained at a load of 3 kΩ, while monitoring the corrosion protection of the anode in various electrolyte environments. Every two days, the spent electrolyte (saline: 2.7 mM KCl and 137 mM NaCl or physiological saline: 20 mM phosphate, 2.7 mM KCl and 137 mM NaCl, pH 7.4) was exchange of for a fresh electrolyte.

The formation of the corrosion protection layer (aluminum hydroxide and phosphite) on the Al/Au/ZnO anode was achieved by discharging the anode in physiological saline using a two-electrode cell configuration (platinum served as the cathode) across 3 kΩ resistor for 10–15 min. Polarization curves were obtained for both bare Al, Al/Au, untreated Al/Au/ZnO, saline treated Al/Au/ZnO and aluminum hydroxide and phosphite coated Al/Au/ZnO anodes in 20 mM phosphate buffer solution at room temperature using a three-electrode cell configuration. Ag/AgCl and platinum electrode were used as the reference and counter electrodes, respectively. The protective abilities of corrosive resistant films formed on the surface of Al/Au/ZnO anode in different electrolyte environments were examined using electrochemical impedance spectroscopy. An a.c. potential of 10 mV_p-p_ was applied over the frequency range of 100 kHz to 10 mHz.

## 3. Results and Discussion

**Figure 1:**
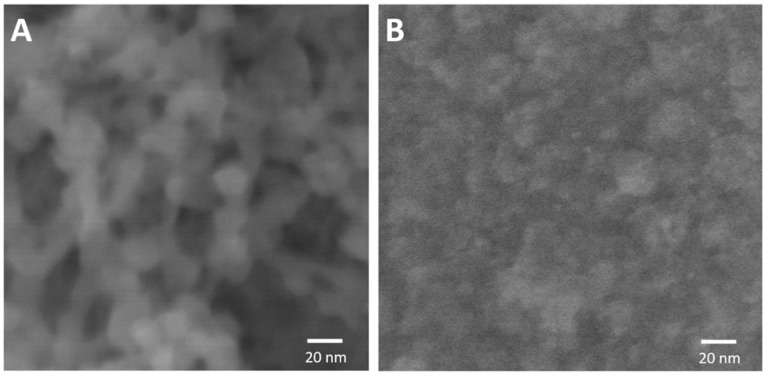
Scanning electron micrograph of Al/Au/ZnO anode (**A**) before and (**B**) after discharging in physiological saline (pH 7.4, 20 mM phosphate, 2.7 mM KCl and 137 mM NaCl) to form nonporous aluminum hydroxide and zinc phosphite lamellae layer on the anode.

**Figure 2:**
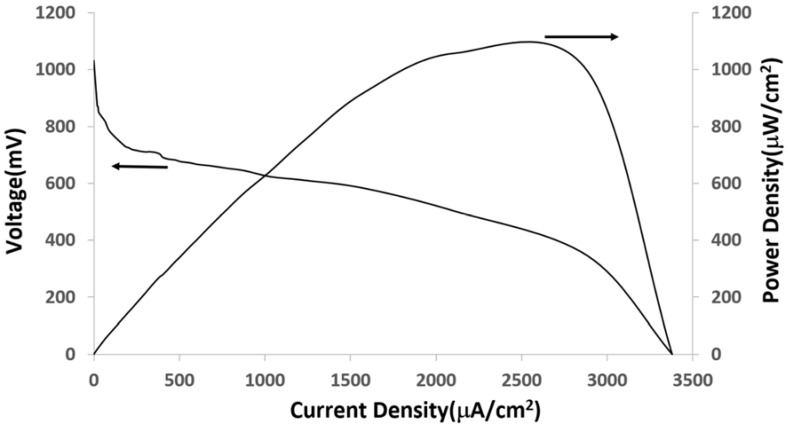
Current-voltage characterization and power impact curves of the hybrid cell in O_2_-saturated phosphate buffered saline.

The as-prepared Al/Au/ZnO anodes (1 cm × 0.5 cm) and platinum cathodes (ϕ = 500 μm) were assembled to realize a hybrid cell operating in physiological saline buffer (pH 7.4) saturated with O_2_. In the hybrid cell, the nanostructured ZnO plugged in on the anodic electrode surface (Figure 1A) results in the Al/Al^3+^ oxidation via pitting mechanism originating from the defect site to overcome the thin oxide film on the Al substrate in physiological saline. The oxidation of the byproduct of the anodic reaction, hydrogen, results in the release of electrons that recombines with the H_2_PO_4_^−^ ions on the surface of the platinum cathode to reduce it to HPO_3_^2−^ ions. These ions then react with Zn^2+^ release from ZnO nanocrystals and the adsorption and precipitation of HPO_3_^2−^ results in the formation of Al(OH)_3_ and ZnHPO_3_ composite on the anode (Figure 1B). The current-voltage and power characteristics were acquired immediately upon assembling the hybrid cell and are shown in Figure 2. This cell achieved a 6-fold increase in performance compared to the previously reported Al/Au/ZnO anode in abiotic fuel cell application [15]. The maximum power density in physiological saline buffer was 955.3 μW/ cm^2^ at a cell voltage of 345 mV with approximately the same open circuit voltage value. This enhancement in performance can be attributed to the increased ZnO nanoseed layer deposited on the Al/Au substrate. The highest maximum power density reported to date is achieved using Al/Au/ZnO anode described here when compared to enzymatic and abiotic fuel cells and biobatteries that use biological fluids as an electrolyte. Briefly, enzymatic fuel cells have the potential to exhibit long life spans but lack in high electrical characteristics when compared with lithium ion cells [37,38]. Although, enzymatic biofuel cells have been reported to last for 3 months, continuous operation was only observed over a period of 9 days [39]. This shortcoming is due to the loss of enzyme activity, thereby resulting in shorter device lifetime. On the other hand, biobatteries and abiotic fuel cells have been able to produce the most comparable electrical properties that may closely match lithium ion cells but have only shown to survive for a few weeks at most [28,31]. The cell described here was able to produce almost 10 times more power than recently reported abiotic fuel cells [40,41], around 5 times more power than enzymatic biofuel cells [31,42,43,44,45], 2 to 6 times more power than biobattery that uses a zinc phosphate anode [28,31] and has a 1.25 times more current density that of the traditional lithium ion cell [46,47].

**Figure 3:**
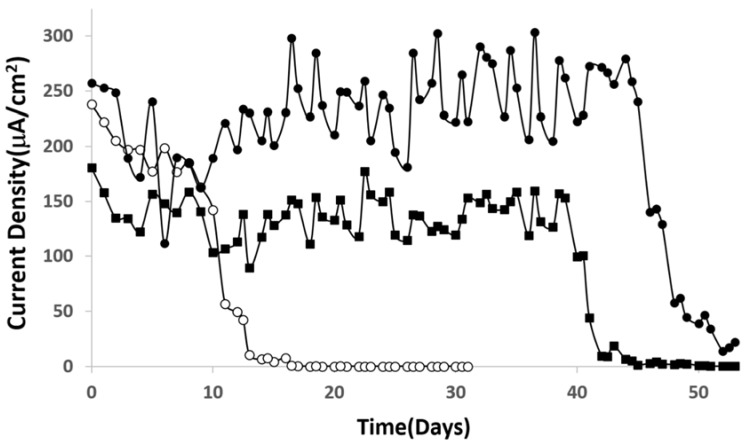
Stability curve of the hybrid cell continuously operating in O_2_-saturated (○) phosphate buffer, (▪) saline, and (●) physiological saline buffer environments.

The durability of the cells employing Al/Au/ZnO anodes and platinum cathodes were operated continuously under a 3 kΩ load in all three separate electrolyte environments are depicted in Figure 3. Polarization curves were obtained once a day for 55 days, with the electrolyte environment being exchanged with fresh electrolyte every two days. Overall, the results show good durability and stability of voltage and current density. Upon operating the cell in saline solution, significant deterioration of the anode was observed. The Al/Au/ZnO anode in this environment was immediately discolored, followed by the appearance of pits in week 4. In week 6, the anode was completely corroded, which led to the complete decomposition of the Al/Au/ZnO anode. In this saline environment, the Cl^−^ ions deteriorate the oxide layer on the anode, resulting in a lack of corrosion protection of the Al/Au/ZnO anode. However, the current density of the cell operating in phosphate buffer dropped significantly after 10 days of continuous operation, whereas the cell operating in physiological saline rich solution maintained its stability over 40 days of operation. The addition of a halide salt such as NaCl may contribute to the formation of nonporous aluminum hydroxide and zinc phosphite lamellae layer [30] on the surface of the anode in physiological saline that provides corrosion protection of the A/Au/ZnO anode because the film acts as non-oxidizing anode inhibitors, thereby preventing galvanic corrosion of the anode and ionic current flow. The formation of the composite lamellae layer further inhibits power generation during long term durability testing.

**Figure 4:**
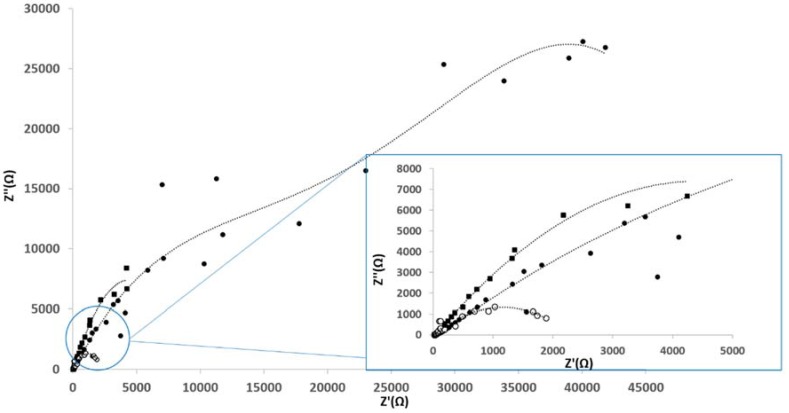
Nyquist plots of (○) untreated Al/Au/ZnO, (_▪_) saline treated Al/Au/ZnO, and (●) Al(OH)_3_ and ZnHPO_3_ coated Al/Au/ZnO anodes.

**Figure 5:**
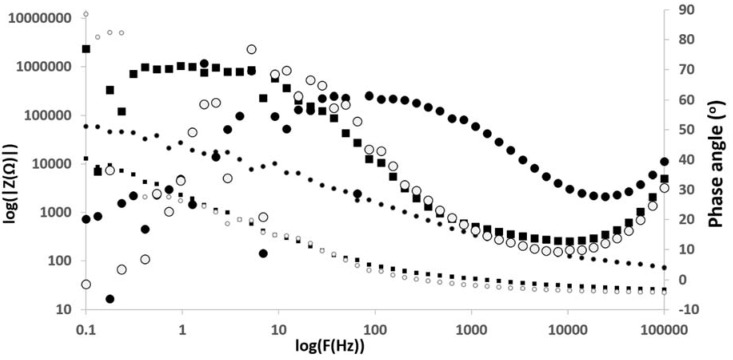
Impedance *vs.* Frequency plots and Bode phase plots for (○) untreated Al/Au/ZnO, (_▪_) saline treated Al/Au/ZnO, and (_●_) Al(OH)_3_ and ZnHPO_3_ coated Al/Au/ZnO anodes.

**Figure 6:**
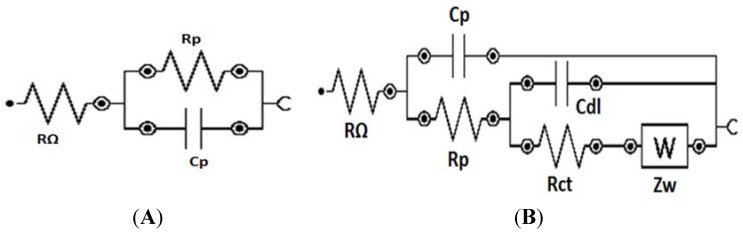
Equivalent electrical circuit utilized to model the impedance behavior of (**A**) saline treated Al/Au/ZnO and (**B**) Al(OH)_3_ and ZnHPO_3_ coated Al/Au/ZnO anodes.

Electrochemical impedance spectroscopy (EIS) was performed to evaluate the inhibitive properties of the corrosion protection composite films. Nyquist and Bode phase angle plots of Al/Au/ZnO anodes (1 cm × 0.5 cm) treated in saline and physiological saline electrolyte environments for a period of 55 days are shown in Figure 4 and Figure 5. The typical electrical equivalent circuit obtained for coated metal surfaces exposed to corrosive environments [48] agree with the electrical equivalent circuit for the Al/Au/ZnO anodes presented in Figure 6. Figure 6A represent the equivalent circuit model for Al/Au/ZnO metal/ electrolyte interface, where R_Ω_ is the solution resistance, R_p_ is the polarization resistance, and C_p_ is the coating capacitance. The equivalent circuit shown in Figure 6B, with the Warburg element (Z_w_), represents the Al/Au/ZnO anode coated with aluminum hydroxide and zinc phosphite composite film upon discharging in physiological saline, where R_ct_ is the charge transfer resistance and C_dl_ is the double layer capacitance. This anode exhibited very small high frequency capacitive loop and is nearly shielded by a diffusion tail in the low frequency domain, thereby showing Warburg behavior. The Nyquist and Bode plots for the coated anode are different from those of the saline treated anode. The high frequency capacitive loop in the Nyquist plot is evident in the saline treated Al/Au/ZnO anode. In the Bode plot, the maximum phase angle is found to be 67.1° and is shifted towards the low frequency region. These results reveal the absence of a corrosion protective film on the saline treated anodic surface. One time constant was observed for the saline treated anode, which suggest that it could only influence the charge transfer process. The |Z| *vs.* f curves also shows a decrease in impedance values for the saline treated anode, which suggest the formation of conductive paths in the ZnO film is due to water uptake and the penetration of corrosive Cl^−^ ions that deteriorates the ZnO layer and the anodic substrate.

The aluminum hydroxide and zinc phosphite coated anode exhibited a Warburg behavior, which indicate the inhomogeneity of corrosion protection film on the anode surface. This film aids in the prevention of the diffusion of oxygen or Cl^−^ ions to the Al substrate and thus inhibits the corrosion of the anode, while enabling the transport of Zn^2+^ in the ZnO conductivity layer. A larger partial semicircle is observed from high frequency to low frequency indicating that the charge transfer resistance is dominant in the corrosion process due to the presence of corrosion protection film on the anode. This is also supported by the Bode plots, in which the phase angle maximum is slightly decreased (61°) and shifted towards higher frequency region compared to the saline treated anode. At anode surfaces coated with aluminum hydroxide and zinc phosphite upon discharging physiological saline environment, the phase angle maximum shows two time constants. The maximum in the low frequency domain relates to the porous ZnO films on the Al surface and the second maximum in the higher frequency domain relates to the charge transfer resistance by the corrosion protection film. The |Z| *vs.* f curves shows an increase in impedance values, which infers that the protective surface film is a combination of zinc oxide film, aluminum hydroxide and zinc phosphite [15]. The formation of aluminum hydroxide and zinc phosphite on the Al/Au/ZnO anode results in a larger charge transfer resistance, R_ct_, value 20.8 kΩ cm^2^, whereas smaller values of 4.48 and 4.17 kΩ cm^2^ was observed for the saline treated and the phosphate buffer treated anodes, respectively. The double layer capacitance, C_dl_, value of 1.50 µF cm^−2^ for the aluminum hydroxide and zinc phosphite coated anode is lower than that of 48.3 and 32.5 µF cm^−2^ observed for the saline treated and phosphate buffer treated anodes, respectively. An admittance of the constant phase element of the double layer was only observed for the aluminum hydroxide and zinc phosphite coated anode, which can be attributed to the formation of a densely packed dielectric film or an increase of the double layer thickness. The high R_ct_ and lower C_dl_ values of the composite film on Al/Au/ZnO anode discharged in physiological saline reveal the dense protective film formation on the surface of Al/Au/ZnO anode.

**Figure 7:**
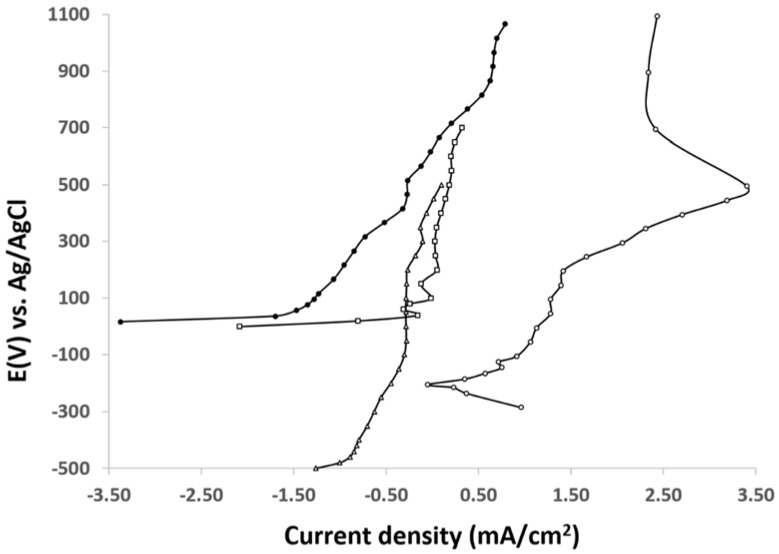
Polarization curves for (∆) bare Al, (□) bare Al/Au, (○) untreated Al/Au/ZnO, and (●) Al(OH)_3_ and ZnHPO_3_ coated Al/Au/ZnO anodes.

In addition, the Al/Au/ZnO anodes were examined after the 55 days discharge in saline, phosphate buffer and physiological saline environments. The polarization behavior of the Al/Au/ZnO anodes are depicted in Figure 7. The bare Al/Au anode shows higher anodic activity than that of the bare Al anode, suggesting the Al/Au is more inert than the bare Al. The corrosion potential (E_corr_) and corrosion current density (i_corr_) can be determined using the Tafel extrapolation method [49] and are approximated with the position of the anodic current in the polarization curves. The activation of Al via nano-ZnO seeds deposited on the surface of Al/Au anode allow for zinc ions to react with phosphite groups to form aluminum hydroxide and zinc phosphite composite in phosphate buffer and physiological saline buffer [15]. The Al/Au/ZnO anode coated with this protective film shifts the potential of the anode to become more anodic and thus decreases corrosion current and enhances the electrical properties. This shift toward more anodic values allows for an increased deposition of the corrosion protection film [50], which further suggests an increased corrosion resistance. The protective efficiency P(%) of the Al/Au/ZnO anode coated with aluminum hydroxide and zinc phosphite composite film was determined by [51]:

$$P\left(\%\right)=100\;\left(1-\frac{K_{f}}{K_{f}^{0}}\right)$$

where $K_{f}$ and $K_{f}^{0}$ are the faradaic conductances in the presence and absence of the composite film, respectively. Here the protective efficiency of 98.5% was calculated for the Al/Au/ZnO anode coated with aluminum hydroxide and zinc phosphite composite film. The polarization behavior of the Al/Au/ZnO anode coated with this protective film demonstrates the effective corrosion protection of the anode.

The aluminum hydroxide and zinc phosphite composite films are impermeable to O_2_ and thus prevent corrosion [28,30] even in the presence of NaCl for over one month and allow for increased cell performance. Therefore, the cell operating in physiological saline buffer resulted in the most durable cell, lasting up to 50 days. The observed life span of the Al/Au/ZnO anode in physiological saline is much longer that previously reported abiotic fuel cells [28,29,30]. The enhanced durability of the cell operating in physiological saline buffer suggests that localized sites of aluminum hydroxide and zinc phosphite composite film enable the development of micro regions on the surface of the anode to allow Zn^2+^ ions to react with phosphites to form corrosion protection film while reducing the galvanic current [52]. Thereby, resulting in an improved corrosion resistance when operating in physiological saline buffer.

## 4. Conclusion

The corrosion protection of the as-fabricated Al/Au/ZnO anode was characterized in saline, phosphate rich buffer and physiological saline buffer. The Al/Au/ZnO anode discharged in phosphate rich buffer and physiological saline buffer showed protection against corrosion via the formation of aluminum hydroxide and zinc phosphite films. The resulting corrosion resistant anode led to improved electrical properties (current density) and extended life span of the hybrid fuel cell when compared to abiotic fuel cells based on similar anodes. The use of the sol–gel dip coating process yielded a much larger surface area of zinc oxide, which was employed to activate the aluminum anode. The biomimetic approach employed in this work is more economical and environmentally safe compared to other techniques that require harmful nitrates, nitrites, acidic baths and high energy equipment. Here, we demonstrate a novel approach to providing corrosion resistant films on Al/Au/ZnO anodes while harvesting energy. The cell exhibited an enhanced performance and extended lifetime over other abiotic fuel cells. The excellent performance of the cell can be attributed to the combined effect of the cathode and the Al/Au/ZnO anode that operated in physiological saline buffer to achieve an open circuit voltage of 1.03 V. The Al/Au/ZnO has significant potential for developing long lasting hybrid cells.
